# Favorable Lithium Nucleation on Lithiophilic Framework Porphyrin for Dendrite-Free Lithium Metal Anodes

**DOI:** 10.34133/2019/4608940

**Published:** 2019-01-06

**Authors:** Bo-Quan Li, Xiao-Ru Chen, Xiang Chen, Chang-Xin Zhao, Rui Zhang, Xin-Bing Cheng, Qiang Zhang

**Affiliations:** Beijing Key Laboratory of Green Chemical Reaction Engineering and Technology, Department of Chemical Engineering, Tsinghua University, Beijing 100084, China

## Abstract

Lithium metal constitutes promising anode materials but suffers from dendrite growth. Lithiophilic host materials are highly considered for achieving uniform lithium deposition. Precise construction of lithiophilic sites with desired structure and homogeneous distribution significantly promotes the lithiophilicity of lithium hosts but remains a great challenge. In this contribution, a framework porphyrin (POF) material with precisely constructed lithiophilic sites in regard to chemical structure and geometric position is employed as the lithium host to address the above issues for dendrite-free lithium metal anodes. The extraordinary lithiophilicity of POF even beyond lithium nuclei validated by DFT simulations and lithium nucleation overpotentials affords a novel mechanism of favorable lithium nucleation to facilitate uniform nucleation and inhibit dendrite growth. Consequently, POF-based anodes demonstrate superior electrochemical performances with high Coulombic efficiency over 98%, reduced average voltage hysteresis, and excellent stability for 300 cycles at 1.0 mA cm^−2^, 1.0 mAh cm^−2^ superior to both Cu and graphene anodes. The favorable lithium nucleation mechanism on POF materials inspires further investigation of lithiophilic electrochemistry and development of lithium metal batteries.

## 1. Introduction

The ever growing demand of energy supply stimulates endless prusuit of high-performance energy storage devices [1]. Lithium (Li) metal with an ultrahigh theoretical specific capacity of 3860 mAh g^−1^ and the lowest electrochemical potential of −3.040 V versus the standard hydrogen electrode constitutes a promising anode material to construct high-energy-density batteries [2, 3]. However, the electrochemistry of Li metal is intrinsically a hostless process with virtually infinite volume change and drastic morpholgy variation during Li plating and stripping [4, 5]. The morphology variation inevitably results in the crack/repair of solid electrolyte interphase (SEI) that continuously consumes the electrolyte and induces low Coulombic efficiency (CE) [6, 7]. In addition, uneven Li deposition causes the formation of notorious Li dendrites [8]. The Li dentrites not only breed “dead Li” with fast capacity decay, but also raise potential safety hazards such as internal short circuit that hinders the practical applications of Li metal batteries [9, 10].

Introducing a host material as a stable second phase to accommodate Li deposition offers a reasonable strategy to address the above hostless issues [11]. Three-dimensional (3D) porous copper [12], fibrous Li_7_B_6_ [13], and ZnO composites [14] exhibit evident improvements to suppress the dendritic formation as Li hosts. In particular, three-dimensional carbon materials have drawn worldwide attentions as favorable Li hosts for dendrite-free Li metal anodes [15]. Large surface area of 3D carbon materials significantly lowers the local current density to inhibit Li dendrites [16]. Advantages of high electronic conductivity, excellent mechanical and chemical stability, and low density additionally promote the potential of carbon materials as Li host candidates. For instance, Cui and coworkers reported hollow carbon nanospheres as Li hosts for stable Li plating and stripping [17]. Porous carbon networks [18], spherical carbon granules [19], and crumpled graphene balls [20] are also proved effective in regulating uniform Li deposition. Nevertheless, the nonpolar nature of carbon materials affords poor affinity with the polar Li species. High nucleation barrier and sluggish kinetics of Li deposition induce uncontrollable Li nucleation and growth under electrochemical polarization conditions. Eventually, messy Li dendrites cut off the lifespan of Li metal batteries. Therefore, endowing the Li hosts with excellent lithiophilicity to improve their compatibility with Li species constitutes the key issue to fabricate stable dendrite-free Li metal anodes.

As an analogous concept of hydrophilia, lithiophilicity is defined as the capability of a material to form a stable structure with Li [21]. Pioneer researches reported by Yan et al. demonstrate the lithiophilicity of gold (Au) to form alloys with Li, and hollow carbon shells modified with Au nanoparticles exhibit selective Li deposition on lithiophilic Au rather than conductive carbon matrix [22]. Silica microspheres [23], zinc clusters [24], and heteroatom doped graphene [25] also demonstrate good lithiophilicity to regulate Li deposition. However, precise construction of lithiophilic sites in regard to their chemical structure and geometrical position at the atomic level remains a grand challenge [26]. Once an absolute homogeneous distribution of lithiophilic sites is achieved, the lithiophilicity would be driven to extreme and inspires innovation in lithiophilic electrochemistry for dendrite-free Li metal anodes.

Herein, a framework porphyrin (POF) material with precise lithiophilic sites in regard to chemical structure and geometrical position is introduced as the lithiophilic host to facilitate uniform Li nucleation for high-performance dendrite-free Li metal anodes. POF is constructed by covalently linking porphyrin units into extended two-dimensional (2D) frameworks ([Supplementary-material supplementary-material-1]) [27]. The porphyrin units are planar, polar, and highly conjugated with four electron-rich pyrrolic nitrogen atoms serving as the lithiophilic sites [25]. The covalent linkages with intrinsic directionality and saturability guarantee the absolute configuration of the predesigned POF at the atomic level to render homogeneous distribution of the lithiophilic sites [28, 29]. When employed as the host material for Li deposition, POF reveals extraordinary lithiophilicity that demonstrates novel Li nucleation behavior and new mechanism of favorable Li nucleation.

## 2. Results and Discussion

**Figure 1:**
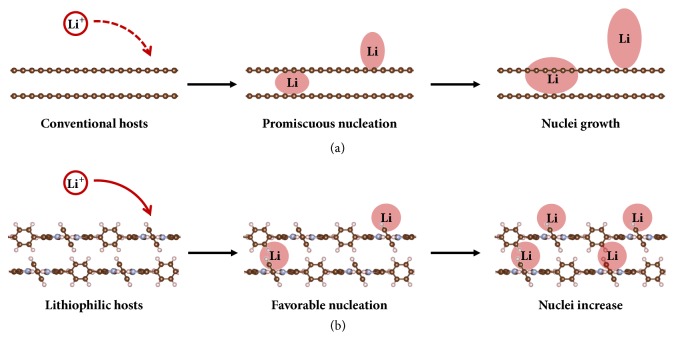
**Schematic of Li nucleation on host materials with different lithiophilicities.** (a) Conventional hosts with poor affinity of Li render promiscuous nucleation and dendrite growth. (b) Favorable Li nucleation on lithiophilic hosts with precisely constructed lithiophilic sites to afford dendrite-free Li metal anodes.

The schematic of favorable Li nucleation on lithiophilic POF is illustrated in Figure 1. Conventional hosts (for instance, most carbon materials) with poor affinity with Li ions require high overpotential to provide extra energy for Li deposition. The reduction of Li ions is spatially nonuniform under electrochemical polarization conditions, and Li nucleation is accordingly uneven. Compared with nonpolar carbon hosts, the as-generated polar Li nuclei are more lithiophilic to function as prepotent nucleation sites. Therefore, the Li nuclei grow in size afterwards ascribed to the routine nucleation–growth mechanism. Although the carbon materials afford large surface area for initial Li nucleation, their deficiency of lithiophilicity limits subsequent restriction of nuclei growth into dendrites. Consequently, Li metal anodes using conventional hosts come to failure at high current densities or during long-term cycling.

In contrast, POF functions as a lithiophilic host that extensively attract Li ions to its surface. Affinity of POF with Li ions significantly lowers the nucleation barrier and facilitates initial Li nucleation. Compared with the as-generated Li nuclei, POF is surprisingly more lithiophilic that Li ions prefer to deposit on POF rather than Li nuclei proposed as the favorable nucleation mechanism. Accordingly, the Li nuclei increase in amount but maintain in size to render homogeneous Li nucleation. Therefore, subsequent smooth Li plating and dendrite-free Li metal anodes can be achieved.

**Figure 2:**
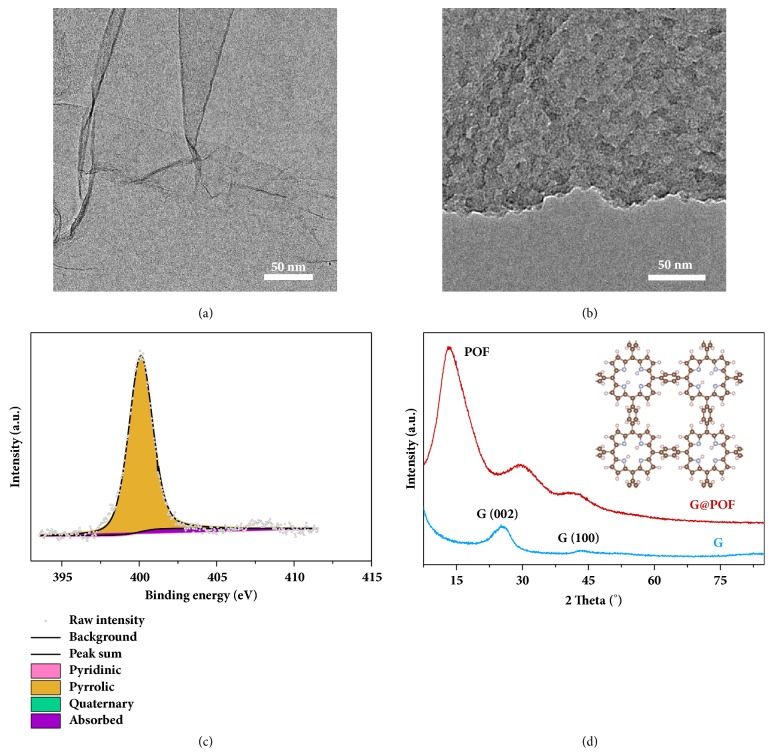
**Characterization of G@POF.** TEM images of (a) G and (b) G@POF. (c) High-resolution nitrogen 1s XPS spectrum and (d) XRD patterns of G@POF. The insert in (d) is the chemical structure of POF.

POF was one-pot synthesized following the direct synthesis methodology as the lithiophilic host. Graphene (named as G) was introduced as the template to avoid the stacking of POF layers and increase the overall conductivity, and the hybrid of G and POF was named as G@POF. Compared with neat G sheets characterized using scanning electron microscopy (SEM) and transmission electron microscopy (TEM) (Figures 2(a) and [Supplementary-material supplementary-material-1]), G@POF exhibits a distinct morphology of G wrapped in POF ([Supplementary-material supplementary-material-1]). TEM images (Figures 2(b) and [Supplementary-material supplementary-material-1]) further demonstrate the homogeneous coating of POF layers on G sheets with each POF flake being* ca.* 20 nm in diameter.

Fourier transformed infrared spectrometry (FTIR) was carried out to evaluate the progress of the POF synthesis reaction. The characteristic adsorption peak of POF precursors at 1700 cm^−1^ is absent while a new adsorption band at 1650 cm^−1^ assigned to the C=N vibration appears ([Supplementary-material supplementary-material-1]) [30], suggesting full conversion of the precursors to the desired POF structure. Element analysis of G@POF indicates an explicit nitrogen content of 10.1 wt.% by the combustion method (COM), which is in agreement with the theoretical nitrogen content of 9.8 wt.% ([Supplementary-material supplementary-material-1]). Further X-ray photoelectron spectroscopy (XPS) analysis ([Supplementary-material supplementary-material-1]) and energy-dispersive X-ray spectrometer (EDS) results ([Supplementary-material supplementary-material-1]) confirm the reasonable nitrogen content of 6.7 and 10.1 at.%, respectively, serving as a side evidence. Suitable nitrogen content promises potential lithiophilicity of G@POF.

Precise fabrication and homogneneous distribution of the desired lithiophilic sites contribute significantly to the lithiophilicy of host materials. High-resolution nitrogen 1s XPS spectrum in Figure 2(c) indicates the lithiophilic pyrrolic nitrogen with a portion of 93.6% as the dominant nitrogen species with negligible pyridinic or quaternary nitrogen [31, 32]. Therefore, the nitrogen species of G@POF are sufficiently lithiophilic to maximize the lithiophilicity of G@POF as the Li host. On the other hand, X-ray diffraction (XRD) patterns of G@POF reveal a characteristic diffraction peak at 13° (Figure 2(d)), suggesting the intrinsic ordered structure of POF. Such ordered structure of the predesigned porphyrin units linked into 2D layers guarantees the homogeneous distribution of lithiophilic sites from aggregation, further amplifying the lithiophilicity of POF. Element mapping additionally confirms uniform distribution of nitrogen in G@POF ([Supplementary-material supplementary-material-1]).

The specific surface area of G@POF (482.1 m^2^ g^−1^) is lower than G (604.1 m^2^ g^−1^) but maintains in the same order of magnitude ([Supplementary-material supplementary-material-1](a)). The 1.3 nm micropore of G@POF is exclusive as an evidence of the POF structure with similar mesopores afforded by G ([Supplementary-material supplementary-material-1](b)).

**Figure 3:**
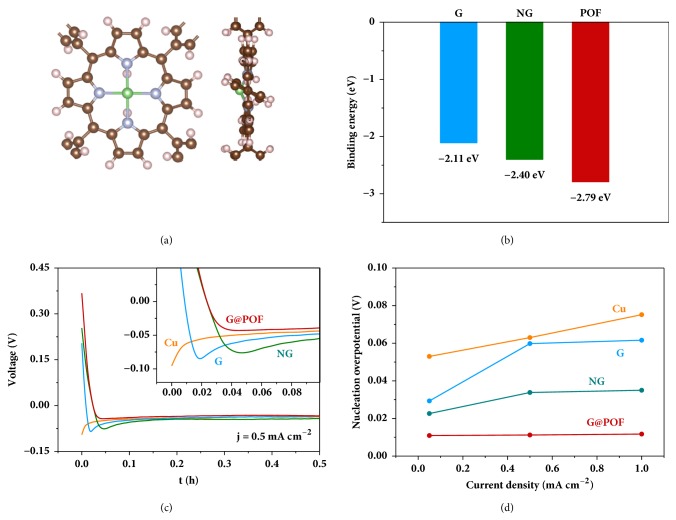
**Evaluation of Li nucleation.** (a) Optimized geometrical structures of Li binding to POF from the top view and the side view. The hydrogen, lithium, carbon, and nitrogen atoms are marked with white, green, brown, and blue, respectively. (b) Binding energy of Li with G, NG, and POF. (c) Voltage–time curves of Li nucleation at the current density of 0.50 mA cm^−2^ and (d) Li nucleation overpotentials at different current densities on Cu, G, NG, and G@POF electrodes.

One typical signature of the lithiophilic hosts is the capability of forming stable structures with Li species. Specifically, lithiophilic hosts are proposed to competitively attract solvated Li ions to generate advantageous configurations. Theoretical simulations based on the density functional theory (DFT) was performed to evaluate the affinity of Li with host materials of G, conventional nitrogen-doped G (named as NG), and POF (Figure 3(a) and [Supplementary-material supplementary-material-1]). POF affords the largest binding energy of −2.79 eV beyond G (−2.11 eV) and even NG (−2.40 eV) with predominant lithiopihlicity (Figure 3(b)). Further differential charge density analysis indicates that the highly conjugated structure of porpyrin plays an important role in strong electronic interactions with Li through intermolecular polarization ([Supplementary-material supplementary-material-1]). The as-formed Li–N bond is further validated by the N 1s XPS spectrum ([Supplementary-material supplementary-material-1]), in which the peak at 399.1 eV is identified as the Li–N interaction signal [33].

Li nucleation overpotential at the initial stage of Li deposition is selected as the experimental descriptor to evaluate the lithiophilicity of host materials [34]. As expected, G@POF demonstrates the lowest nucleation overpotential of 14.6 mV at the current density of 0.50 mA cm^−2^ (Figure 3(c)), and the superiority is inherited at other current densities (Figures 3(d) and [Supplementary-material supplementary-material-1]). Notably, the lithiophilic order obtained from the experiments is in good agreement with the simulation results, conclusively confirming the excellent lithiophilicity of POF.

**Figure 4:**
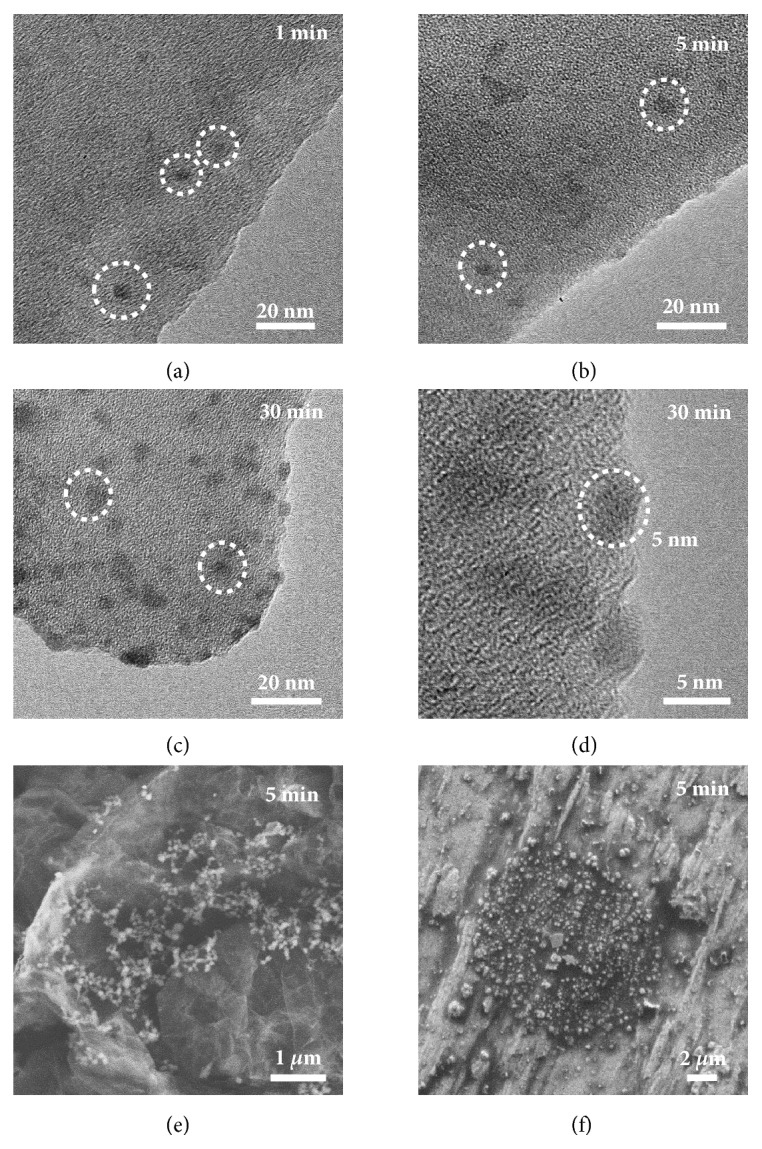
**Morphology evolution of Li nucleation on lithiophilic hosts.** TEM images of G@POF after Li deposition at the current density of 0.50 mA cm^−2^ for (a) 1 min, (b) 5 min, (c) and (d) 30 min. SEM images of (e) G and (f) Cu after Li deposition at the current density of 0.50 mA cm^−2^ for 5 min.

The final morphology and performance of Li metal anodes are largely dependent on the initial Li nucleation behavior [25]. Time-dependent morphology characterization was carried out to investigate the morphology evolution on lithiophilic hosts. Galvanostatic Li plating was performed at 0.50 mA cm^−2^ using the electrolyte without LiNO_3_ additive to reveal the lithiophilic nature of Li hosts. After 1 min Li deposition, dot contrast was observed and assigned as Li nuclei with a diameter of* ca.* 5 nm (Figures 4(a) and [Supplementary-material supplementary-material-1]). More Li nuclei were generated with higher deposition capacity but the size of the Li nuclei remained unchanged (Figures 4(b) and [Supplementary-material supplementary-material-1]). When the deposition duration increased to 30 min, the Li nuclei amount increased sharply while the size of the Li nuclei surprisingly remained similar to the diameter around 5 nm without obvious nuclei growth (Figures 4(c), 4(d), and [Supplementary-material supplementary-material-1]). Such nucleation behavior suggests that POF possesses higher affinity of Li ions than Li nuclei to perform as favorable deposition sites and therefore verifies the novel mechanism of favorable nucleation on lithiophilic POF. In contrast, aggregated Li nuclei were found on lithiophobic G and Cu, which is detrimental to following Li deposition (Figures 4(e), 4(f), and [Supplementary-material supplementary-material-1]).

The development of the as-generated Li nuclei was monitored with higher deposition capacity. After Li plating at 0.50 mA cm^−2^ for 1.0 h, plenty Li dendrites were observed on Cu ([Supplementary-material supplementary-material-1]) while G and G@POF were free of Li dendrites (Figures [Supplementary-material supplementary-material-1] and [Supplementary-material supplementary-material-1]). When the Li plating time increased to 4.0 h, Cu and G suffered from severe dendrite growth (Figures [Supplementary-material supplementary-material-1] and [Supplementary-material supplementary-material-1]). To our satisfaction, G@POF survived to afford a dendrite-free morphology throughout the process ([Supplementary-material supplementary-material-1]), which is attributed to the excellent lithiophilicity and favorable Li nucleation.

**Figure 5:**
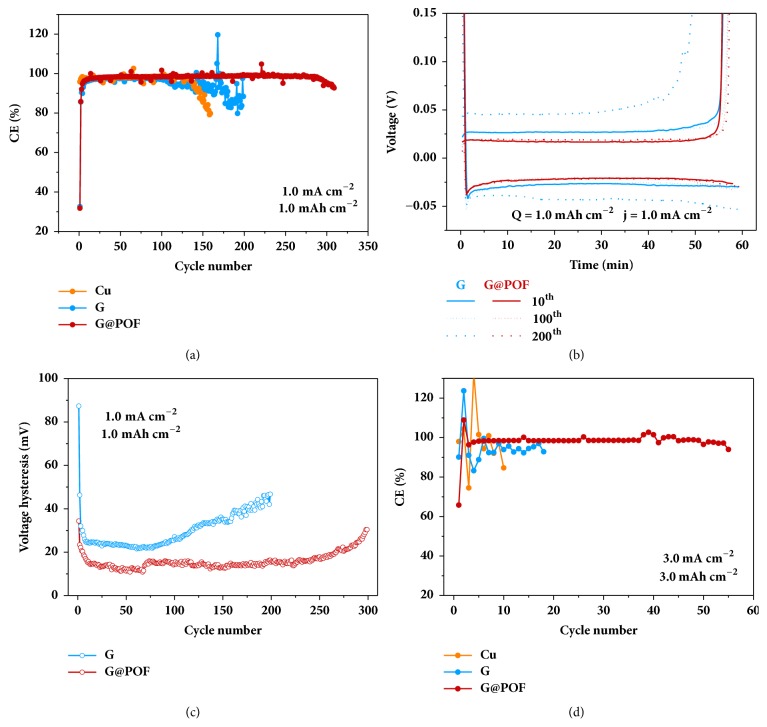
**Electrochemical performance.** (a) CE of Cu, G, and G@POF electrodes at the current density of 1.0 mA cm^−2^ and the capacity of 1.0 mAh cm^−2^. (b) Corresponding voltage profiles of the 10^th^, 100^th^, and 200^th^ cycle and (c) average voltage hysteresis of the G and G@POF electrodes. (d) CE of Cu, G, and G@POF electrodes at the current density of 3.0 mA cm^−2^ and the capacity of 3.0 mAh cm^−2^.

The uniform Li nucleation and dendrite-free morphology of G@POF encourage further electrochemcial evaluation in working conditions. Two-electrode cells were assembled using a Li metal foil as the counter electrode and Cu, G, and G@POF electrodes as the working electrode. The cells were first cycled at the current density of 1.0 mA cm^−2^ and the capacity of 1.0 mAh cm^−2^. The G@POF electrode ran stably for 300 cycles with the CE retaining over 98% while Cu and G electrodes failed after 160 and 200 cycles, respectively (Figure 5(a)). The voltage hysteresis represents the electrochemical polarization degree for Li plating and stripping [23]. Voltage profiles in Figure 5(b) demonstrate that the voltage hysteresis remained under 20 mV for G@POF but increased dramatically in cases of G. The reduced and stable voltage hysteresis of G@POF is further demonstrated in Figure 5(c), suggesting faster kinetics and favored Li reactions. Both the porphyrin nitrogen and the organic framework structure of POF contribute to the excellent performance of the G@POF electrode.

At higher cycling current density of 2.0 mA cm^−2^ and capacity of 2.0 mAh cm^−2^, the G@POF electrode afforded robust CE over 98% for 150 cycles while the CE of Cu and G electrodes decayed rapidly halfway ([Supplementary-material supplementary-material-1]). The G@POF electrode even stood for 50 cycles at 3.0 mA cm^−2^, 3.0 mAh cm^−2^ with the CE above 95%. On the contrary, Cu and G electrodes disabled within 20 cycles under identical conditions (Figures 5(d) and [Supplementary-material supplementary-material-1]). Considering comparable specific surface area and similar electrolyte ohmic resistance of G and G@POF ([Supplementary-material supplementary-material-1]), the lithiophilicity of POF affords the decisive contribution toward superior electrochemical performance of Li metal anodes.

## 3. Conclusion

In conclusion, a framework porphyrin (POF) material was rationally designed, fabricated, and employed as the lithiophilic host material for dendrite-free Li metal anodes. Precise structure fabrication and homogeneous distribution of porphyrin lithiophilic sites were achieved on POF materials. The extraordinary lithiophilicity of POF even beyond Li nuclei affords the novel mechanism of favorable Li nucleation to render uniform Li deposition from dendrite growth. Consequently, POF-based lithium metal anodes demonstrate superior electrochemical performances with reduced voltage hysteresis, high Coulombic efficiency over 98%, and satisfactory lifespan for 300 cycles. The favorable Li nucleation mechanism on POF materials with exceeded lithiophilicity not only affords rational design principles for lithiophilic hosts to inhibit lithium dendrite growth, but also inspires further investigation of lithiophilic electrochemistry and development of Li metal batteries.

## 4. Methods

### 4.1. Raw Materials

Benzene-1,4-dialdehyde (BDA) (98%), pyrrole (99%), trifluoroacetic acid (99%), nitrobenzene (99%), propionic acid (99%), and N-methyl pyrrolidone (NMP) (99%) were purchased from Alfa Aesar Chemical Co., Ltd., and directly used without further purification. Copper foils, Celgard 2400 polypropylene (PP) membranes, and poly(vinylidenefluoride) (PVDF) were purchased from Shenzhen Kejing Star Technology Co., Ltd. Lithium metal foils were purchased from China Energy Lithium Co., Ltd. 1,2-dimethoxyethane (DME) (99%), 1,3-dioxolane (DOL) (99%), lithium nitrate (LiNO_3_) (99.98%), and lithium bis(trifluoromethanesulfonyl)imide (LiTFSI) (98%) were purchased from Alfa Aesar Chemical Co., Ltd., and kept in a glove box.

### 4.2. Synthesis of G, NG, and G@POF

G was fabricated by thermal reduction of graphite oxide in vacuum at high temperature and detailed procedures can be found in our previous works [35, 36].

NG was fabricated by annealing G in NH_3_ atmosphere [37]. Typically, 100 mg G was placed in the middle of a horizontal quartz tube within a furnace. The furnace was heated to 600°C under Ar flow (150 mL min^−1^) with a heating rate of 10°C min^−1^. After the temperature was stable, NH_3_ as the nitrogen source was introduced to the reactor with a flow of 150 mL min^−1^ for 4.0 h. The reactor was then naturally cooled to room temperature under Ar protection, and NG was obtained and directly used without further processing. The pressure was maintained as the atmospheric pressure throughout the annealing and cooling processes.

G@POF was one-pot synthesized using G as the template. Typically, 100 mg G was added into 200 mL propionic acid and the mixture was sonicated for 30 min to afford a homogeneous suspension. 234.3 mg BDA and 242.4 *μ*L pyrrole were then added and the suspension was stirred for 15 min to fully dissolve the precursors. The theoretical mass ratio of G:POF was 1:4. To the suspension was then added 100.0 *μ*L trifluoroacetic acid and 1.0 mL nitrobenzene as the catalyst and the oxidant. The suspension was kept at 130°C for 12.0 h under continuous stirring to complete the reaction. After cooling to room temperature, the product was filtered and washed with ethanol, chloroform, deionized water, and ethanol again for three times, respectively. The purified product was dried at 60°C overnight and 486 mg G@POF was obtained to afford a yield of 97%.

### 4.3. Material Characterization

The morphology of the samples was characterized using a JSM 7401F (JEOL Ltd., Tokyo, Japan) scanning electron microscope (SEM) and a JEM 2010 (JEOL Ltd., Tokyo, Japan) transmission electron microscope (TEM). The operation voltage of SEM and TEM was 3.0 kV and 120.0 kV, respectively. Energy-dispersive X-ray spectrometer (EDS) and corresponding element mapping were performed on the TEM equipped with an Oxford Instrument energy-dispersed X-ray spectrometer. The chemical structure of the samples was evaluated using Fourier-transform infrared spectrometry (FTIR) performed on a NEXUS 870 spectrograph. X-ray diffraction (XRD) was applied to reveal the crystal structure of the samples. The XRD patterns were recorded on a Bruker D8 Advanced diffractometer with Cu-K_*α*_ radiation at 40.0 kV and 120 mA as the X-ray source. Elemental analysis was performed using combustion method (COM) on an Elemental Analyzer (Vario El III, Germany) under O_2_ flow at 1000°C. X-ray photoelectron spectroscopy (XPS) measurements were carried out using Escalab 250xi. The samples were cleaned by argon plasma before measurements. The XPS spectra were corrected using carbon 1s line at 284.6 eV. Nitrogen adsorption-desorption isotherm was recorded using an Autosorb-IQ2-MP-C system at 77 K to characterize the pore structure of the samples. The samples were degassed at 200°C for 10.0 h before physisorption measurements. Specific surface area (SSA) was calculated based on the multipoint Brunauer–Emmett–Teller (BET) methods. Pore-size distribution was determined following the quenched solid density function theory (DFT) model using the data of the adsorption branch.

### 4.4. Electrochemical Evaluation

The electrochemical performance of Cu, G, NG, and G@POF was evaluated using two-electrode cells. Standard CR2025 coin-type cells were employed and all the cells were assembled in an argon-filled glove box with oxygen and water content below 1 ppm.

To prepare the G@POF electrode, G@POF and PVDF were mixed in NMP with a mass ratio of 4:1, and the mixture was stirred for 24.0 h to afford a homogeneous suspension. The suspension was then coated onto a copper foil and dried in vacuum at 60°C for 6.0 h. The thickness of the G@POF layer was about 150 *μ*m and the areal loading of G@POF was 0.437 mg cm^−2^. After the solvent was evaporated, the G@POF coated copper foil was punched into disks with a diameter of 13.0 mm and the as-obtained disks were employed as the working electrode. The fabrication of the G and NG electrodes was otherwise identical to the G@POF electrode except using the same amount of G and NG instead of G@POF, respectively. The Cu electrode was prepared by punching copper foil into 13.0 mm disks which were directly used as the working electrode.

The two-electrode cells were assembled using a Cu, G, NG, or G@POF electrode as the working electrode, a Celgard 2400 PP membrane as the separator, and a lithium metal foil as the counter electrode. The thickness and the diameter of the lithium metal electrode were 0.5 mm and 16.0 mm, respectively. The electrolyte was 1.0 M LiTFSI in DOL/DME mixed solvent (v/v = 1:1) with 5.0% LiNO_3_ additive for the cells. Notably, the cells used for morphology characterization and lithium nucleation tests employed the electrolyte without LiNO_3_ additive to reveal the intrinsic lithiophilicity of the samples.

The assembled cells were monitored in a routine galvanostatic mode using a Land CT2001 multichannel battery tester. In each galvanostatic cycle, the charge time for lithium plating was fixed to afford the given areal capacity while the discharge time for lithium stripping was controlled by a cut-off voltage at 0.5 V without time limitation. The electrochemical impedance spectroscopy (EIS) measurements and lithium nucleation tests with ultralow current density were performed on a Solartron 1470E electrochemical workstation (Solartron Analytical, UK). The EIS data was collected using the as-assembled cells before cycling.

### 4.5. Computational Details

Cluster-based calculations are conducted using the Gaussian (G09) suite of programs; Becke's three-parameter hybrid method using the Lee–Yang–Parr correlation functional (B3LYP) [38] was chosen in this study. Geometries were optimized and vibrational modes were calculated in G09 at B3LYP/6-311++G(d,p) level of theory. The solvation effect was considered with integral equation formalism variant of the Polarizable Continuum (IEFPCM) model [39, 40] as implemented with parameters of dielectric constant *ε* = 7.1/7.2 and solvent radius of 3.71/4.19 Å for DOL/DME, respectively.

The periodic DFT calculations were conducted in Vienna ab initio Simulation package (VASP) [41, 42] with the projector augmented-wave (PAW) [43, 44] pseudopotentials and the results were visualized in VESTA [45]. Perdew–Burke–Ernzerhof (PBE) generalized-gradient approximation (GGA) functional [46] were adopted in all DFT calculations. Particularly, the van der Walls (vdW) interaction was described with DFT-D3 method [47, 48]. The energy cutoff was set to 520 eV. The self-consistent field (SCF) and geometry convergence tolerance were set to 1×10^−5^ and 1×10^−4^ eV, respectively.

A 2×2×1 super cell of single-layer POF with a 1.5-nm vacuum was constructed to interact with lithium polysulfides. For comparisons, a zigzag 6×6×1 graphene nanoribbon (G) with a 20-Å vacuum layer in both the slip direction and normal direction was built. Besides, the pyridine nitrogen doping G (NG) model was also constructed.

The sizes of these models are large enough to avoid the interaction between replicas. A sampling of 1×1×1 and 6×1×1 Monkhorst–Pack k-points [49] was used during the geometrical optimizations for POF and G/NG, respectively.

The binding energy of lithium with POF, G, NG, was defined as follows:

(1) $$\begin{array}{c} E_{b}=E_{total}-E_{slab}-E_{Li} \end{array}$$

where* E*_*total*_,* E*_*slab*_, and* E*_*Li*_ are the total energy of POF/G/NG bound with a Li atom, pristine POF/G/NG, and a Li atom, respectively.

## Data Availability

All data generated or analyzed during this study are included in this published article and its Supplementary Materials.

## Supplementary Materials

Supplementary Materials Figure S1. Schematic of the synthesis routine of POF. The porphyrin units are constructed through proton-activated nucleophilic addition between pyrrole and BDA. The carbonyl groups of BDA in paraconfiguration serve as the knots to connect the porphyrin units through benzene linkages. The structure of POF is intrinsically ordered and two-dimensional. The hydrogen, carbon, nitrogen, and oxygen atoms are marked with white, brown, blue, and red, respectively. Figure S2. Morphology characterization of G. (a) and (b) SEM images and (c) and (d) TEM images of G. Figure S3. SEM images of G@POF at different magnifications. The morphology of G wrapped in POF can be observed, indicating successful hybridization of G and POF. Figure S4. TEM images G@POF at different magnifications. POF flakes are evenly coated on the surface of G sheets with neither POF stacking nor bare G. Figure S5. FTIR spectra of (a) pyrrole and BDA and (b) G and G@POF. The absence of the C=O vibration peak at 1700 cm^−1^ as well as the existence of the C=N vibration peak at 1650 cm^−1^ indicates that the predesigned POF structure is achieved. Figure S6. XPS survey spectra of G and G@POF. G@POF exhibits a promotion in nitrogen content of 6.7 at.% compared with G of 0.4 at.%. Figure S7. EDS patterns of G and G@POF. No nitrogen signal is afforded by G while G@POF demonstrates an explicit nitrogen content of 10.1 at.%. Figure S8. (a) TEM image, (b) dark-field TEM image, and corresponding elemental mapping of (c) carbon and (d) nitrogen of G@POF. The elemental mapping of G@POF indicates uniform distribution of its nitrogen species. Figure S9. (a) Nitrogen sorption isotherms of G and G@POF. The specific surface area calculated using the BET model is 604.1 m^2^ g^−1^ for G and 482.1 m^2^ g^−1^ for G@POF, respectively. (b) Pore size distribution of G and G@POF based on DFT methods. The pore volume of G and G@POF is 2.10 and 1.21 cm^3^ g^−1^, respectively. The distinct 1.3 nm micropore of G@POF is derived from the intrinsic porous structure of POF. Figure S10. Geometry of lithium binding to (a) G and (b) NG from the top view. The hydrogen, carbon, nitrogen, and lithium atoms are marked with white, brown, blue, and green, respectively. Figure S11. Differential charge density analyses of lithium binding to POF from (a) the top view and (b) the side view. The yellow and blue isosurfaces (0.001 |e| Å^−3^) correspond to the charge gain and lost regions, respectively. The hydrogen, carbon, nitrogen, and lithium atoms are marked with white, brown, blue, and green, respectively. Figure S12. High-resolution nitrogen 1s XPS spectra of G@POF after lithium nucleation. A new signal is deconvoluted at 399.1 eV besides the origin pyrrolic nitrogen, which is identified as the interaction between nitrogen and lithium. Figure S13. Voltage–time curves of lithium nucleation at the current density of (a) 0.05 mA cm^−2^ and (b) 1.0 mA cm^−2^ on Cu, G, NG, and G@POF electrodes. The inserts are the corresponding amplified profiles. G@POF demonstrates the lowest nucleation overpotential under both conditions. Figure S14. Morphology characterization of lithium nucleation on the G@POG electrode. (a), (b) and (c) TEM images of G@POF at different magnifications after 1 min lithium deposition at the current density of 0.50 mA cm^−2^. The white circles mark the lithium nuclei with the diameter of* ca.* 5 nm. Figure S15. Morphology characterization of lithium nucleation on the G@POG electrode. (a), (b), and (c) TEM images of G@POF at different magnifications after 5 min lithium deposition at the current density of 0.50 mA cm^−2^. The diameter of the lithium nuclei remained around 5 nm. Figure S16. Morphology characterization of lithium nucleation on the G@POG electrode. (a), (b), and (c) TEM images of G@POF at different magnifications after 30 min lithium deposition at the current density of 0.50 mA cm^−2^. The amount of the lithium nuclei increased evidently while the size of the lithium nuclei remained unchanged. Figure S17. Morphology characterization of lithium nucleation on Cu and G electrodes. SEM images of (a), (b) G and (c), (d) Cu at different magnifications after 5 min lithium deposition at the current density of 0.50 mA cm^−2^. Aggregated lithium nuclei with larger size are explicitly observed on both electrodes. Figure S18. Morphology characterization of lithium deposition on the Cu electrode. SEM images of Cu from (a) the side view and (b), (c) from the top view after lithium deposition for 1.0 h at the current density of 0.50 mA cm^−2^. Plenty lithium dendrites were found. Figure S19. Morphology characterization of lithium deposition on the G electrode. (a) and (b) SEM images of G at different magnifications after lithium deposition for 1.0 h at the current density of 0.50 mA cm^−2^. No obvious lithium dendrites were observed under this condition. Figure S20. Morphology characterization of lithium deposition on the G@POF electrode. (a) and (b) SEM images of G@POF at different magnifications after lithium deposition for 1.0 h at the current density of 0.50 mA cm^−2^. No obvious lithium dendrites were formed and the G@POF electrode remained dendrite-free. Figure S21. Morphology characterization of lithium deposition on the Cu electrode. SEM images of Cu from (a) the side view and (b), (c) from the top view after lithium deposition for 4.0 h at the current density of 0.50 mA cm^−2^. As expected, plenty lithium dendrites were found. Figure S22. Morphology characterization of lithium deposition on the G electrode. (a) and (b) SEM images of G at different magnifications after lithium deposition for 4.0 h at the current density of 0.50 mA cm^−2^. Lithium dendrites were found on the G electrode at such high lithium deposition capacity. Figure S23. Morphology characterization of lithium deposition on the G@POF electrode. (a) and (b) SEM images of G@POF at different magnifications after lithium deposition for 4.0 h at the current density of 0.50 mA cm^−2^. The G@POF electrode maintained dendrite-free throughout the lithium deposition process. Figure S24. Cycling performance of the Cu, G, and G@POF electrodes at the current density of 2.0 mA cm^−2^ and the capacity of 2.0 mAh cm^−2^. (a) Average voltage hysteresis of the G and G@POF electrodes. (b) Coulombic efficiency of the Cu, G, and G@POF electrodes. The G@POF electrode exhibits superior performance with longer cycling life, higher Coulombic efficiency, and reduced voltage hysteresis over the Cu and G electrodes. Figure S25. Average voltage hysteresis of the G and G@POF electrodes at the current density of 3.0 mA cm^−2^ and the capacity of 3.0 mAh cm^−2^. Figure S26. (a) EIS spectra and corresponding simulated results of G and G@POF electrodes. The insert in (a) is the amplified Nyquist plots. The insert in (b) is the equivalent circuit, where R_l_ is the electrolyte resistance, R_ct_ is the charge transfer resistance, CPE is the constant phase element, and Z_W_ is the Warburg impedance, respectively. The subscripts 1 and 2 represent the interface between the working electrode and the electrolyte and the interface between the counterelectrode and the electrode, respectively. The G and G@POF electrodes exhibit similar R_l_ and R_ct2_ but the R_ct1_ of the G@POF electrode is smaller than the G electrode, exhibiting higher Li affinity of G@POF than G.** Table S1. **Composition of G and G@POF.** Table S2. **Relative amount of the nitrogen species of G@POF.** Table S3. **Comparison of the porosity of G and G@POF.
