# Supplementary material for: Favorable Lithium Nucleation on Lithiophilic Framework Porphyrin for Dendrite-Free Lithium Metal Anodes
Source: Research (Wash D C). 2019 Jan 6;2019:4608940. doi: 10.34133/2019/4608940 (PMC6750078; doi:10.34133/2019/4608940)
Supplement: Supplementary Materials — Figure S1. Schematic of the synthesis routine of POF. The porphyrin units are constructed through proton-activated nucleophilic addition between pyrrole and BDA. The carbonyl groups of BDA in paraconfiguration serve as the knots to connect the porphyrin units through benzene linkages. The structure of POF is intrinsically ordered and two-dimensional. The hydrogen, carbon, nitrogen, and oxygen atoms are marked with white, brown, blue, and red, respectively. Figure S2. Morphology characterization of G. (a) and (b) SEM images and (c) and (d) TEM images of G. Figure S3. SEM images of G@POF at different magnifications. The morphology of G wrapped in POF can be observed, indicating successful hybridization of G and POF. Figure S4. TEM images G@POF at different magnifications. POF flakes are evenly coated on the surface of G sheets with neither POF stacking nor bare G. Figure S5. FTIR spectra of (a) pyrrole and BDA and (b) G and G@POF. The absence of the C=O vibration peak at 1700 cm−1 as well as the existence of the C=N vibration peak at 1650 cm−1 indicates that the predesigned POF structure is achieved. Figure S6. XPS survey spectra of G and G@POF. G@POF exhibits a promotion in nitrogen content of 6.7 at.% compared with G of 0.4 at.%. Figure S7. EDS patterns of G and G@POF. No nitrogen signal is afforded by G while G@POF demonstrates an explicit nitrogen content of 10.1 at.%. Figure S8. (a) TEM image, (b) dark-field TEM image, and corresponding elemental mapping of (c) carbon and (d) nitrogen of G@POF. The elemental mapping of G@POF indicates uniform distribution of its nitrogen species. Figure S9. (a) Nitrogen sorption isotherms of G and G@POF. The specific surface area calculated using the BET model is 604.1 m2 g−1 for G and 482.1 m2 g−1 for G@POF, respectively. (b) Pore size distribution of G and G@POF based on DFT methods. The pore volume of G and G@POF is 2.10 and 1.21 cm3 g−1, respectively. The distinct 1.3 nm micropore of G@POF is derived from the intrinsic porous [file 4608940.f1.pdf]

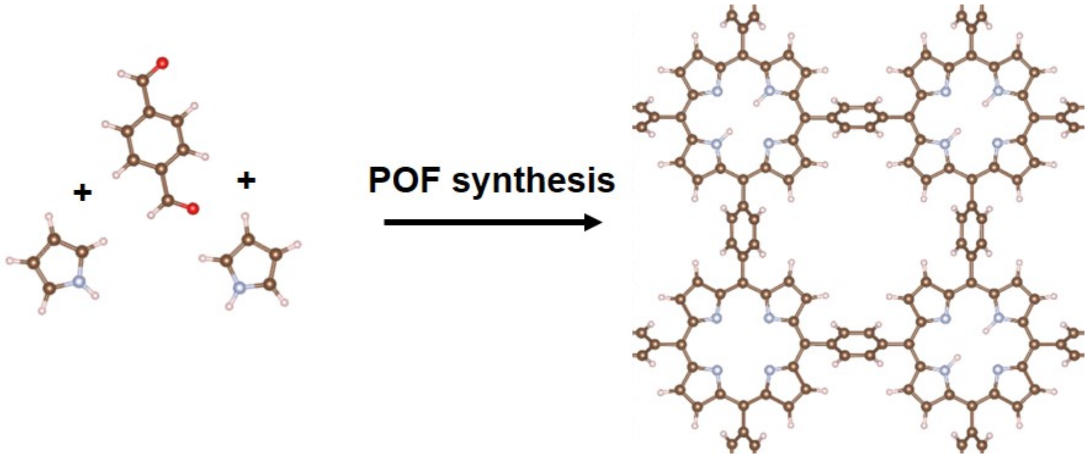

Figure S1. Schematic of the synthesis routine of POF. The porphyrin units are constructed through proton-activated nucleophilic addition between pyrrole and BDA. The carbonyl groups of BDA in para configuration serve as the knots to connect the porphyrin units through benzene linkages. The structure of POF is intrinsically ordered and two-dimensional. The hydrogen, carbon, nitrogen, and oxygen atoms are marked with white, brown, blue, and red, respectively.

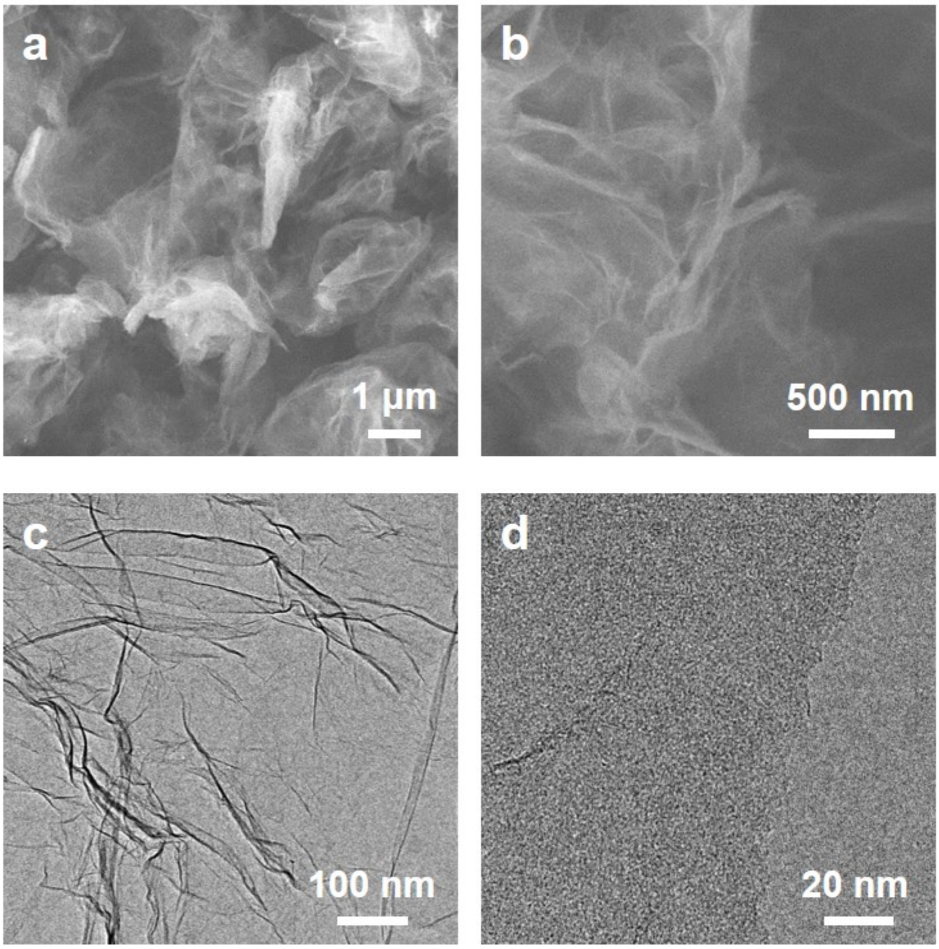

Figure S2. Morphology characterization of G. (a) and (b) SEM images and (c) and (d) TEM images of G.

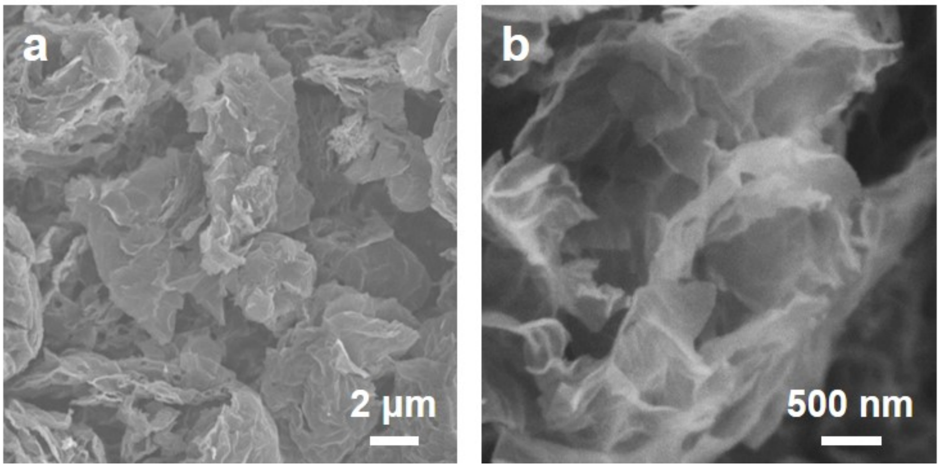

Figure S3. SEM images of G@POF at different magnifications. The morphology of G wrapped in POF can be observed, indicating successful hybridization of G and POF.

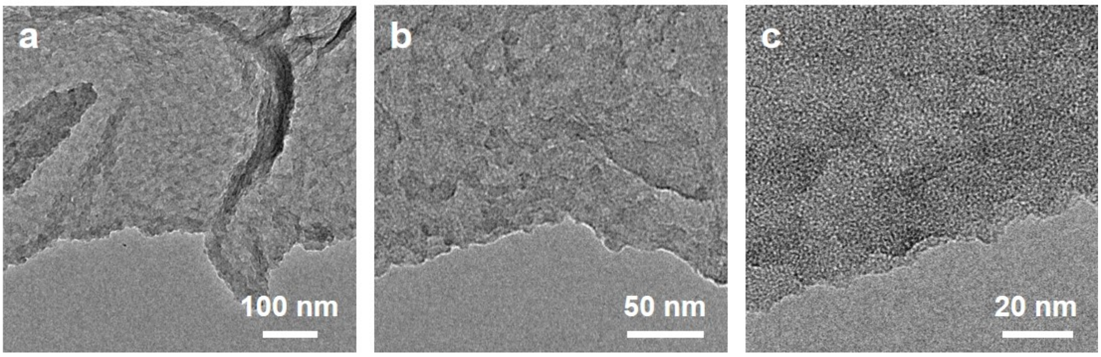

Figure S4. TEM images G@POF at different magnifications. POF flakes are evenly coated on the surface of G sheets with neither POF stacking nor bare G.

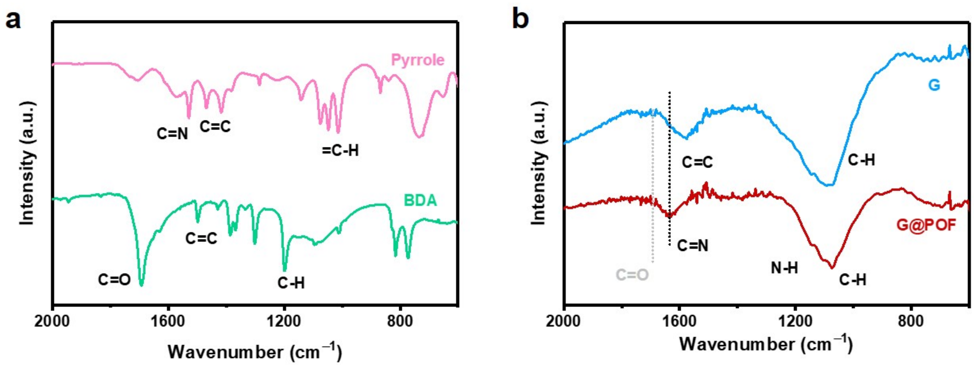

Figure S5. FTIR spectra of (a) pyrrole and BDA and (b) G and G@POF. The absence of the C=O vibration peak at 1700 cm^{-1} as well as the existence of the C=N vibration peak at 1650 cm^{-1} indicate the pre-designed POF structure is achieved.

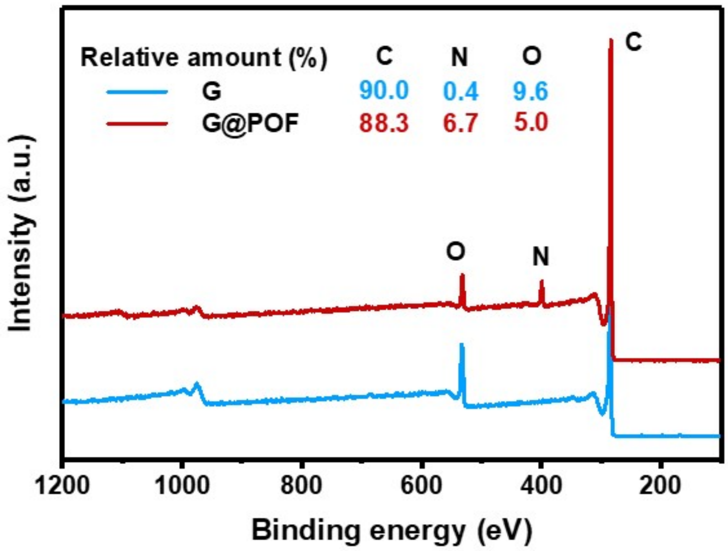

Figure S6. XPS survey spectra of G and G@POF. G@POF exhibits a promotion in nitrogen content of 6.7 at.% compared with G of 0.4 at.%.

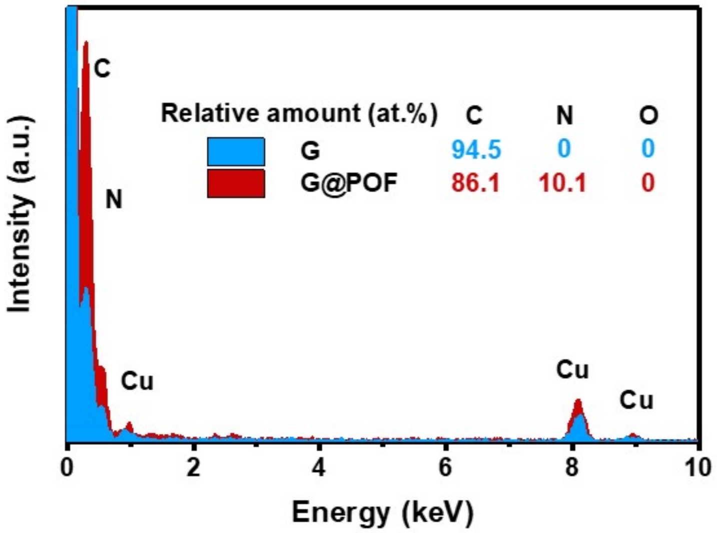

Figure S7. EDS patterns of G and G@POF. No nitogen signal is afforded by G while G@POF demonstrates an explicit nitrogen content of 10.1 at.%.

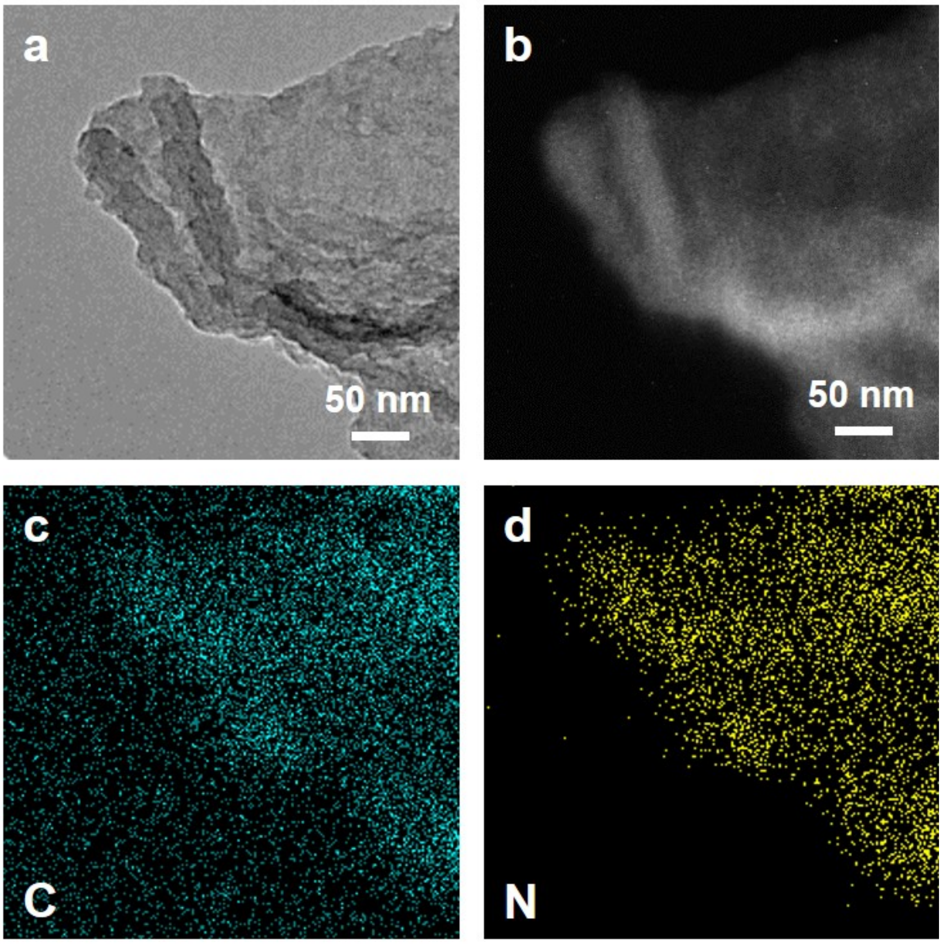

Figure S8. (a) TEM image, (b) dark-field TEM image, and corresponding elemental mapping of (c) carbon and (d) nitrogen of G@POF. The elemental mapping of G@POF indicates uniform distribution of its nitrogen species.

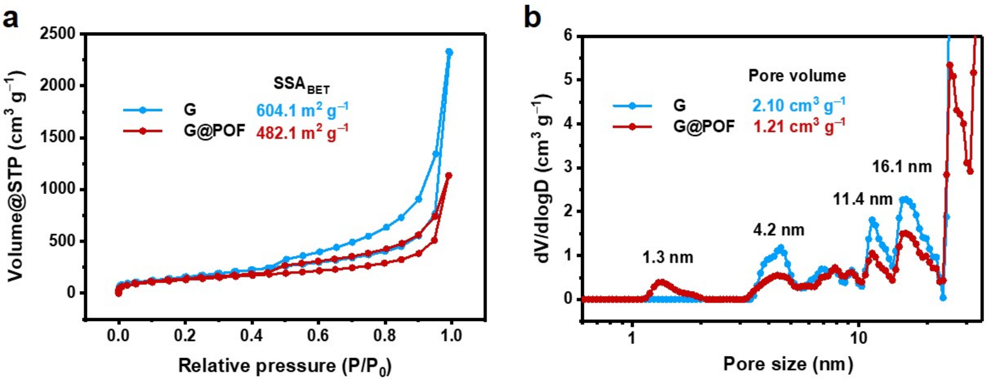

Figure S9. (a) Nitrogen soprtion isotherms of G and G@POF. The specific surface area calculated using the BET model is 604.1 m^2 g^{-1} for G and 482.1 m^2 g^{-1} for G@POF, respectively. (b) Pore size distribution of G and G@POF based on DFT methods. The pore volume of G and G@POF is 2.10 and 1.21 cm^3 g^{-1}, resectively. The distinct 1.3 nm micropore of G@POF is derived from the intrinsic porous structure of POF.

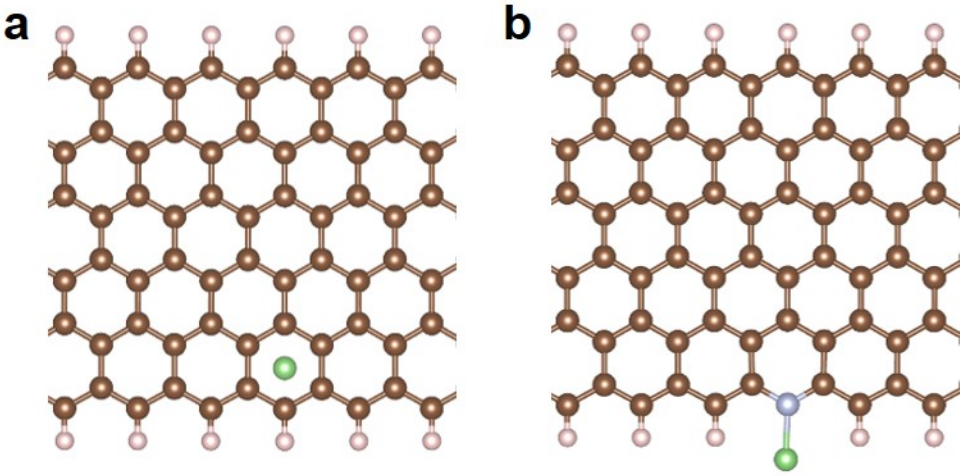

Figure S10. Geometry of lithium binding to (a) G and (b) NG from the top view. The hydrogen, carbon, nitrogen, and lithium atoms are marked with white, brown, blue, and green, respectively.

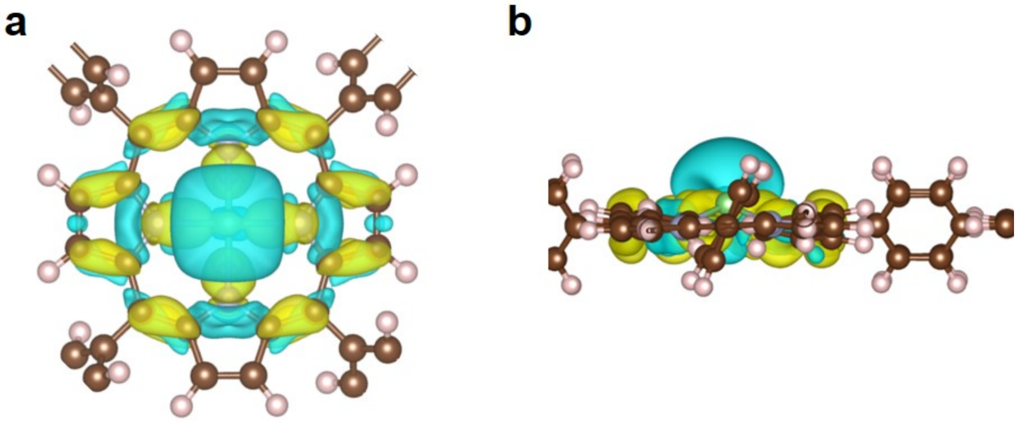

Figure S11. Differential charge density analyses of lithium binding to POF from (a) the top view and (b) the side view. The yellow and blue isosurfaces (0.001 |e| Å^{-3}) correspond the charge gain and lost regions, respectively. The hydrogen, carbon, nitrogen, and lithium atoms are marked with white, brown, blue, and green, respectively.

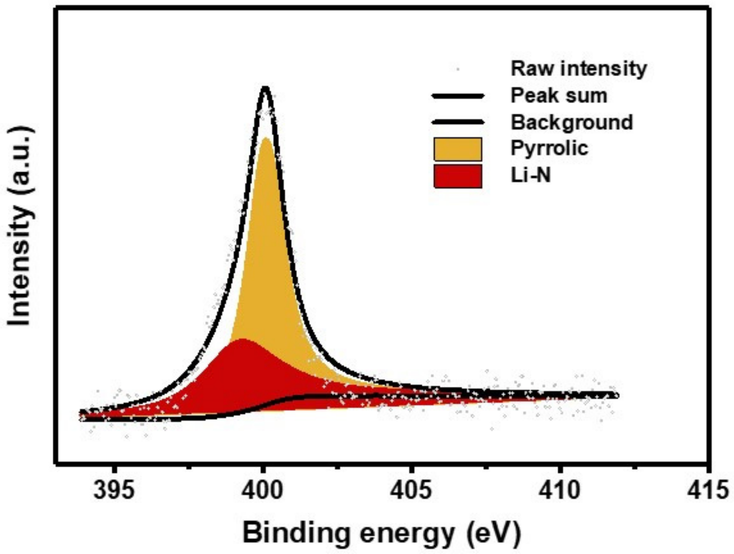

Figure S12. High-resolution nitrogen 1s XPS spectra of G@POF after lithium nucleation. A new signal is deconvoluted at 399.1 eV besides the origin pyrrolic nitrogen, which is identified as the interaction between nitrogen and lithium.

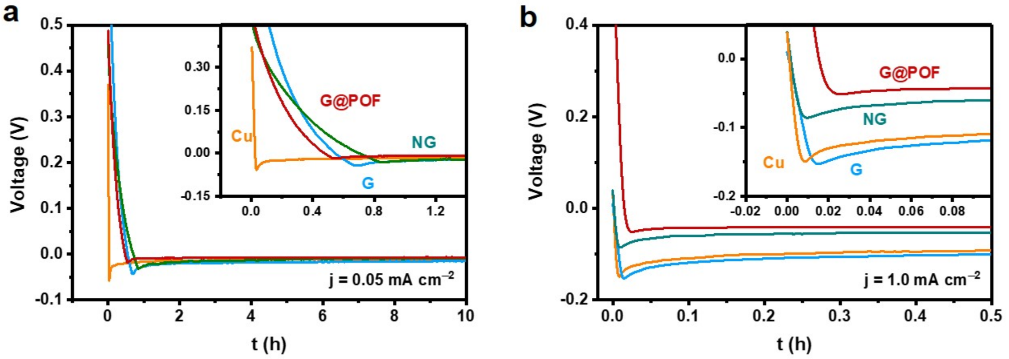

Figure S13. Voltage–time curves of lithium nucleation at the current density of (a) 0.05 mA cm^{-2} and (b) 1.0 mA cm^{-2} on Cu, G, NG, and G@POF electrodes. The inserts are the corresponding amplified profiles. G@POF demonstrates the lowest nucleation overpotential under both conditions.

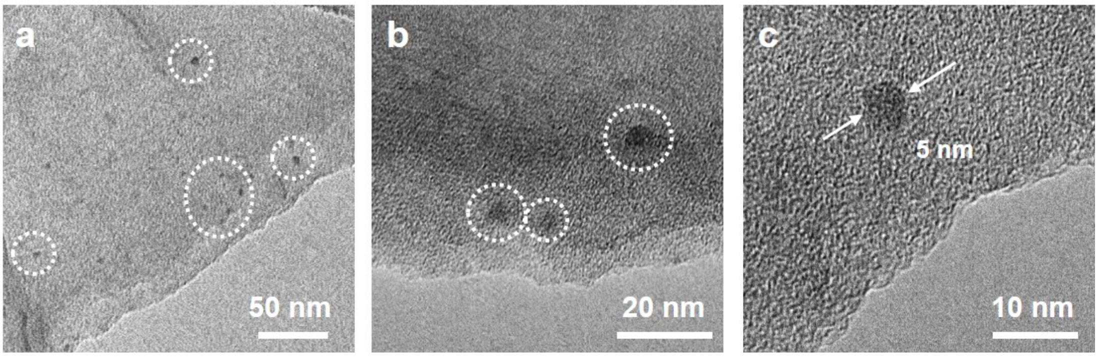

Figure S14. Morphology characterization of lithium nucleation on the G@POG electrode. (a), (b) and (c) TEM images of G@POF at different magnifications after 1 min lithium deposition at the current density of 0.50 mA cm^{-2}. The white circles mark the lithium nucleis with the diameter of *ca.* 5 nm.

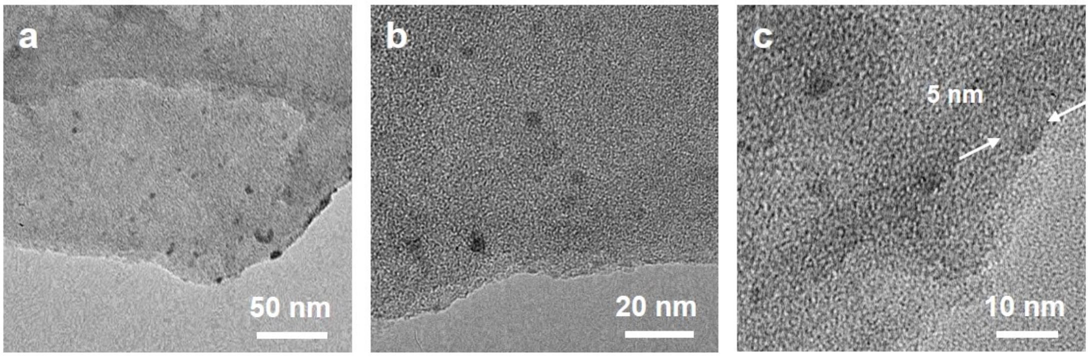

Figure S15. Morphology characterization of lithium nucleation on the G@POG electrode. (a), (b) and (c) TEM images of G@POF at different magnifications after 5 min lithium deposition at the current density of 0.50 mA cm^{-2}. The diameter of the lithium nucleis maintained anound 5 nm.

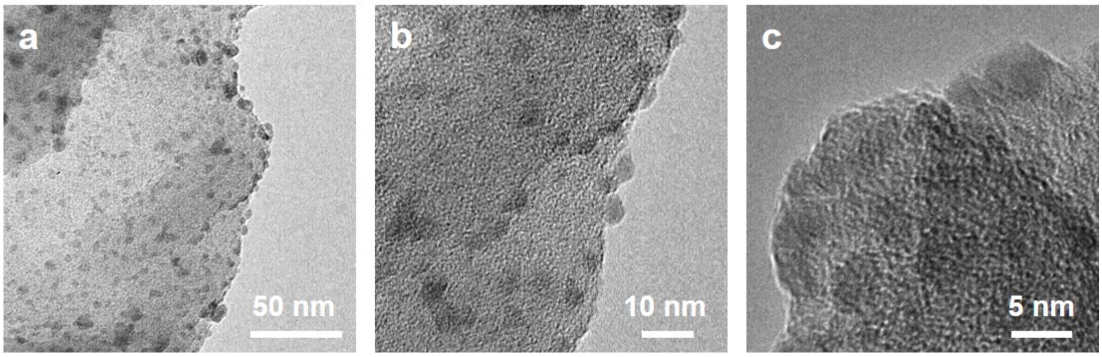

Figure S16. Morphology characterization of lithium nucleation on the G@POG electrode. (a), (b) and (c) TEM images of G@POF at different magnifications after 30 min lithium deposition at the current density of 0.50 mA cm^{-2}. The amount of the lithium nucleis increased evidently while the size of the lithium nucleis remained unchanged.

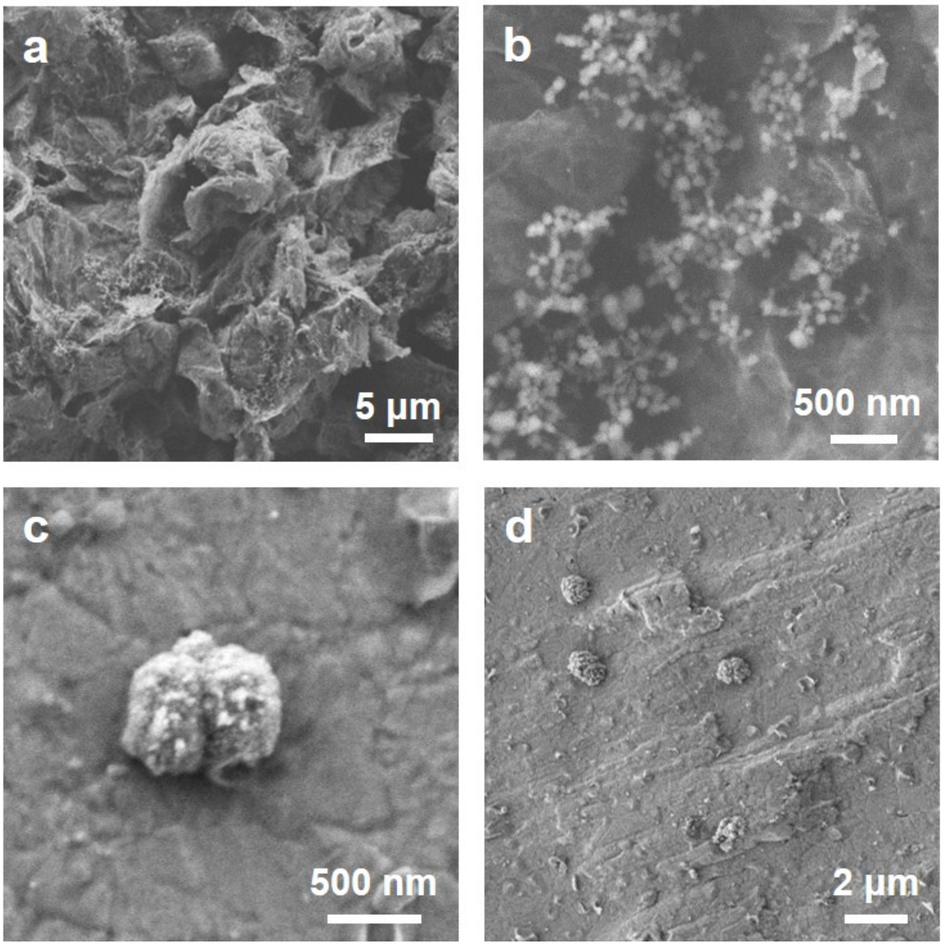

Figure S17. Morphology characterization of lithium nucleation on Cu and G electrodes. SEM images of (a), (b) G and (c), (d) Cu at different magnifications after 5 min lithium deposition at the current density of 0.50 mA cm^{-2}. Aggregated lithium nucleis with larger size are explicitly observed on both electrodes.

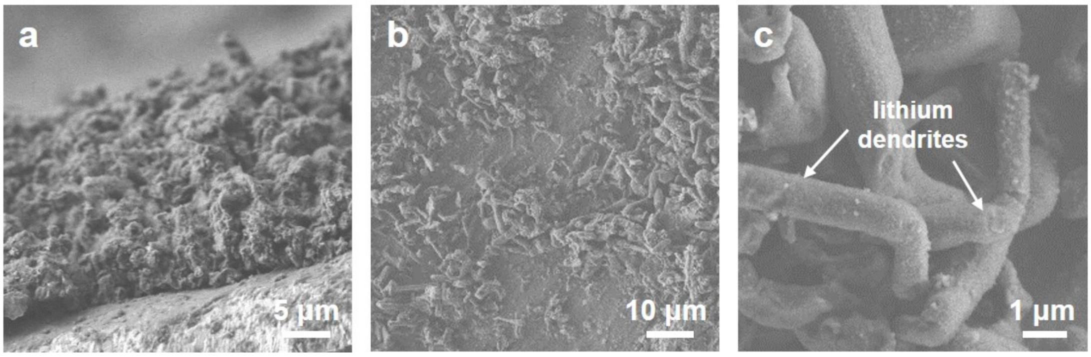

Figure S18. Morphology characterization of lithium deposition on the Cu electrode. SEM images of Cu from (a) the side view and (b), (c) from the top view after lithium deposition for 1.0 h at the current density of 0.50 mA cm^{-2}. Plenty lithiunm dendrites were found.

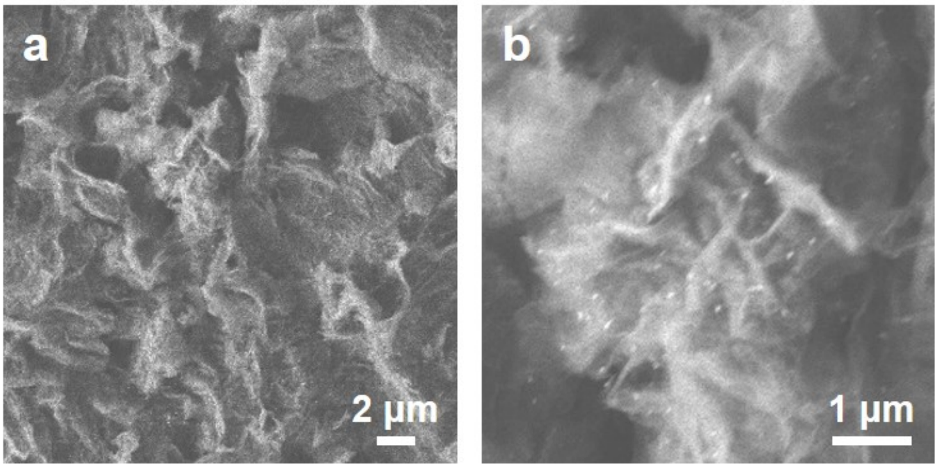

Figure S19. Morphology characterization of lithium deposition on the G electrode. (a) and (b) SEM images of G at different magnifications after lithium deposition for 1.0 h at the current density of 0.50 mA cm^{-2}. No obvious lithium dendrites were observed under this condition.

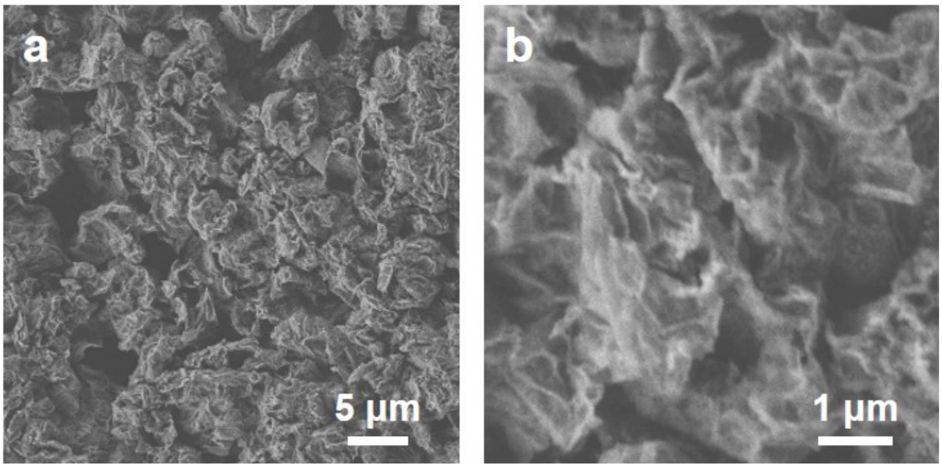

Figure S20. Morphology characterization of lithium deposition on the G@POF electrode. (a) and (b) SEM images of G@POF at different magnifications after lithium deposition for 1.0 h at the current density of 0.50 mA cm^{-2}. No obvious lithium dendrites were formed and the G@POF electrode remained dendrite-free.

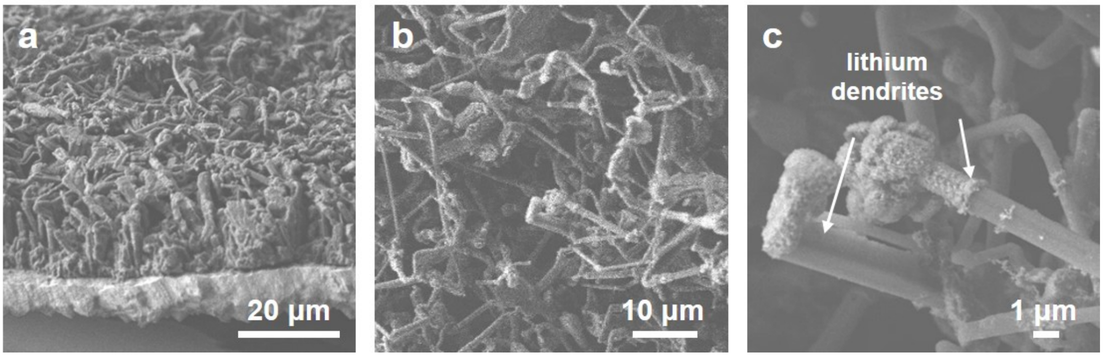

Figure S21. Morphology characterization of lithium deposition on the Cu electrode. SEM images of Cu from (a) the side view and (b), (c) from the top view after lithium deposition for 4.0 h at the current density of 0.50 mA cm^{-2}. As expected, plenty lithium dendrites were found.

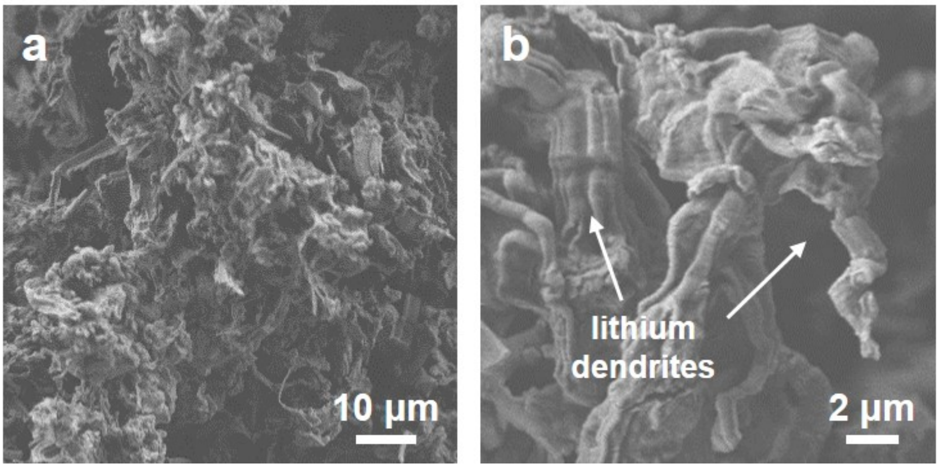

Figure S22. Morphology characterization of lithium deposition on the G electrode. (a) and (b) SEM images of G at different magnifications after lithium deposition for 4.0 h at the current density of 0.50 mA cm^{-2}. Lithium dendrites were found on the G electrode at such high lithium deposition capaticy.

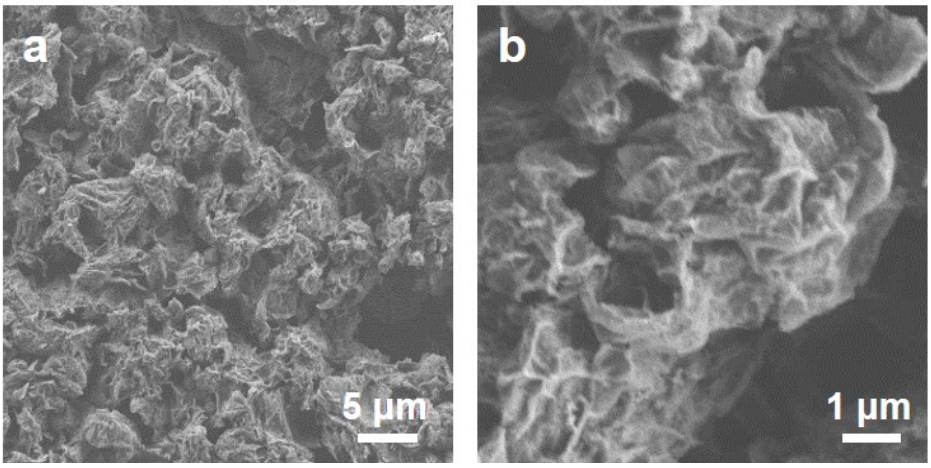

Figure S23. Morphology characterization of lithium deposition on the G@POF electrode. (a) and (b) SEM images of G@POF at different magnifications after lithium deposition for 4.0 h at the current density of 0.50 mA cm^{-2}. The G@POF electrode maintained dendrite-free throughout the lithium deposition process.

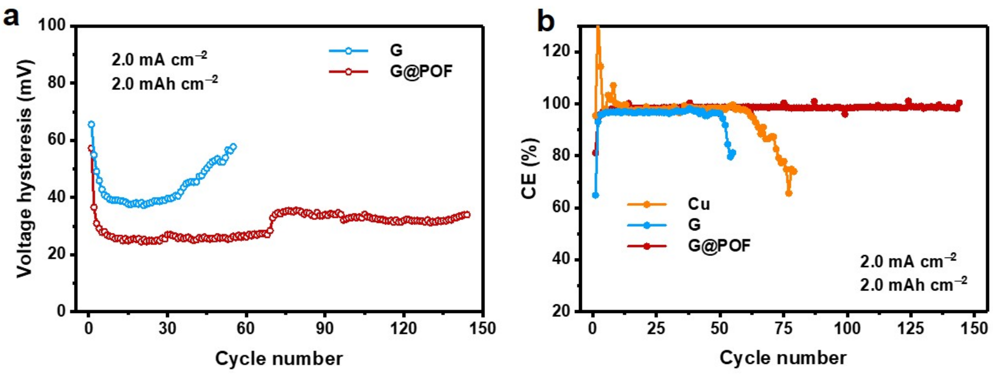

Figure S24. Cycling performance of the Cu, G, and G@POF electrodes at the current density of 2.0 mA cm^{-2} and the capacity of 2.0 mAh cm^{-2}. (a) Average voltage hysteresis of the G and G@POF electrodes. (b) Coulombic efficiency of the Cu, G, and G@POF elecrodes. The G@POF electrode exhibits superior performance with longer cycling life, higher Coulombic efficienty, and reduced voltage hysteresis over the Cu and G electrodes.

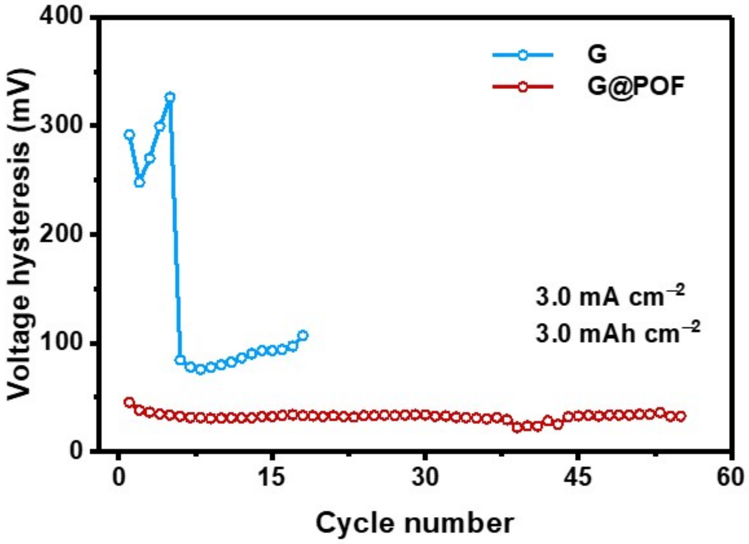

Figure S25. Average voltage hysteresis of the G and G@POF electrodes at the current density of 3.0 mA cm^{-2} and the capacity of 3.0 mAh cm^{-2}.

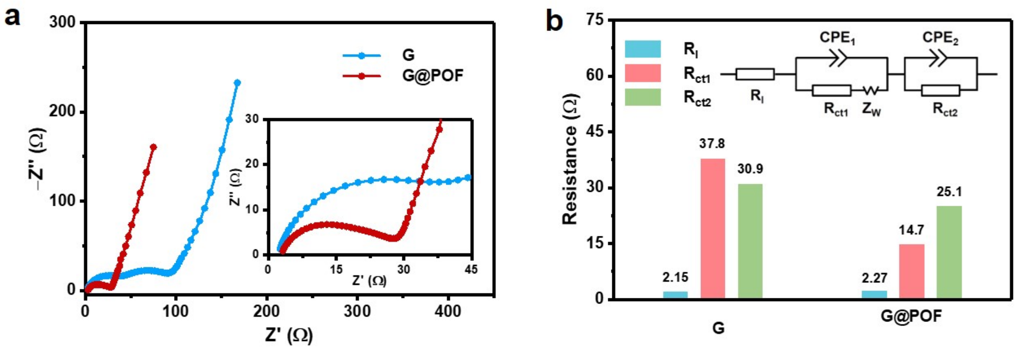

Figure S26. (a) EIS spectra and correspongding simulated resluts of G and G@POF electrodes. The insert in (a) is the amplified Nyquist plots. The insert in (b) is the equicalent circuit, where R_l is the electrolyte resistance, R_{ct} is the charge transfer resistance, CPE is the constant phase element, and Z_W is the Warburg impedance, respectively. The subscripts 1 and 2 represent the interface between the working electrode and the electrolyte and the interface between the counter electrode and the electrode, respectivey. The G and G@POF electrodes exhibit similar R_l and R_{ct2} but the R_{ct1} of the G@POF electrode is smaller than the G electrode, exhibiting higher Li affinity of G@POF than G.

Table S1. Composition of G and G@POF.

Sample	Method	Relative amounts of elements (%)			
		Carbon	Nitrogen	Oxygen	Hydrogen
G	XPS	90.0	0.4	9.6	N.A.
	EDS	94.5	0.0	0.0	N.A.
	COM	98.9	0.2	N.A.	0.9
G@POF	XPS	88.3	6.7	5.0	N.A.
	EDS	86.1	10.1	0.0	N.A.
	COM	86.2	10.1	N.A.	3.7

The unit of the relative amounts of elements is wt.% when using the COM method but at.% when using XPS and EDS.

Table S2. Relative amount of the nitrogen species of G@POF.

Sample	G@POF	
Relative amounts of nitrogen species (at.%)	Pyridinic nitrogen (398.5 eV)	6.4
	Pyrrolic nitrogen (400.1 eV)	93.6
	Quaternary nitrogen (401.2 eV)	0.0
	Absorbed nitrogen (405.6 eV)	0.0

Table S3. Comparison of the porisity of G and G@POF.

Sample	SSA (m^2 g^{-1})	Pore volume (cm^3 g^{-1})
G	604.1	2.10
G@POF	482.1	1.21
